# Immunomodulatory Effects of Plant Extracts from *Salvia deserta* Schang. and *Salvia sclarea* L.

**DOI:** 10.3390/plants11202690

**Published:** 2022-10-12

**Authors:** Aizhan Zhussupova, Gaziza Zhumaliyeva, Vyacheslav Ogay, Assel Issabekova, Samir A. Ross, Galiya E. Zhusupova

**Affiliations:** 1 Department of Molecular Biology and Genetics, NPJSC Al-Farabi Kazakh National University, Al-Farabi Ave. 71, Almaty 050040, Kazakhstan; 2 Stem Cell Laboratory, National Center for Biotechnology, Qorghalzhyn Highway 13/5, Astana 010000, Kazakhstan; 3 School of Pharmacy, University of Mississippi, P.O. Box 1848, University, MS 38677, USA; 4 School of Pharmacy, S.D. Asfendiyarov Kazakh National Medical University, Almaty 050000, Kazakhstan; 5 Department of Chemistry and Technology of Organic Substances, Natural Compounds and Polymers, NPJSC Al-Farabi Kazakh National University, Al-Farabi Ave. 71, Almaty 050040, Kazakhstan

**Keywords:** sage, immunomodulatory, conventional extraction, ultrasonic-assisted extraction

## Abstract

Medicines, their safety, effectiveness and quality are indispensable factors of national security, important on a global scale. The COVID-19 pandemic has once again emphasized the importance of improving the immune response of the body in the face of severe viral infections. Plants from the *Salvia* L. genus have long been used in traditional medicine for treatment of inflammatory processes, parasitic diseases, bacterial and viral infections. The aim of the current study was to evaluate the immunomodulatory effects of plant extracts LS-1, LS-2 from *Salvia deserta* Schang. and LS-3, LS-4 from *Salvia sclarea* L. plants growing in southern Kazakhstan by conventional and ultrasonic-assisted extraction, respectively. The cytotoxic effects of the named sage extracts on neonatal human dermal fibroblasts (HDFn) were evaluated using the MTT assay. Immunomodulatory effects of the studied extracts were compared by examining their influence on pro-inflammatory cytokine secretion and phagocytic activity of murine immune cells. Depending on the physiological state of the innate immune cells, sage extracts LS-2 and LS-3 had either a stimulating effect on inactivated macrophages or suppressed cytokine-producing activity in LPS-activated macrophages. The greatest increase in TNF-α secretion was found after treatment of spleen T lymphocytes with sage extract LS-2, obtained by ultrasonic-assisted extraction.

## 1. Introduction

The importance of the quality of medicinal products for national security was once again highlighted at the 74th World Health Assembly in 2021, with improved and more equitable access to health products designated as a global priority. Herbal drugs, due to their similarity with the physiological systems of the body, are capable of purposefully inducing and mobilizing its protective resources. The advantage of herbal drugs is in the mildness and complexity of their therapeutic action, low toxicity, lack of cumulative effect, addiction, and rare induction of allergic reactions, which is especially important in the case of diseases requiring long-term treatment. For some diseases, herbal drugs may be the only cure. Subsequently, a clear tendency towards an increase in the share of herbal drugs in the total amount of manufactured medicines in leading world countries can obviously be traced, and this figure has now exceeded 50% [1].

It is known from the Sixth National Report of the Republic of Kazakhstan (RK) on biological diversity that the wild-growing flora of the country are represented by 5754 species of higher vascular plants and up to 14% of endemic plants. More than 700 plant species are mentioned in traditional medicine, of which 263 have been studied on a scientific level. Reserves of the overwhelming majority of available medicinal plants, if they are expediently procured, would be sufficient to meet the needs of medicine in RK; however, at the moment, only 5% of them are industrially elaborated [2].

There are some parameters which need to be considered for the procurement of such, including their biological safety, absence of allergic and cumulative properties, therapeutic activity and value, their availability in the territory of the country, conditions of cultivation, complexity and profitability of technology for obtaining complexes of biologically active compounds (BAC) on their basis as well as preservation of ecosystem uniqueness during their procurement [3]. Based on the results of in-depth literature review on all of the aforesaid parameters, the above is true for plants of the genus *Salvia* (sage) of the Lamiaceae family.

Plants from the named genus have been used for a long time in traditional medicine. In Asia and Latin America, its most known representative, *Salvia (S.) officinalis* L., has been used for the treatment of different kinds of disorders, including seizures, ulcers, gout, rheumatism, inflammation, dizziness, tremors, paralysis, diarrhea, and hyperglycemia; and in Europe, to treat mild dyspepsia, excessive sweating, age-related cognitive disorders, and inflammations in the throat and skin [4].

Phenolic compounds, such as carnosol, carnosic and rosmarinic acids, rosmadial, rosmanol, epirosmanol, methyl carnosate, and luteolin-7-O-β-D-glucopyranoside, with high antioxidant activity, are usually extracted from sage with ethanol [5]. It is thought that they can either stimulate endogenous antioxidant defense systems or scavenge reactive oxygen species [6]. In addition, salvianolic acid L, which is a rosmarinic acid dimer isolated from the sage extract, showed a high antioxidant activity and is a very significant scavenger of free radicals [7]. Rosmarinic acid, a natural compound with very low toxicity, which could potentially be used as a therapeutic agent in the treatment of Alzheimer’s disease, as a component of sage, has shown neuroprotective, antioxidant, and anti-apoptotic effects against amyloid beta plaques toxicity, and this could contribute, at least in part, to the neuroprotective effect of sage [8]. Methyl carnosate, carnosic acid, carnosol, rosmanol and salvianolic acids contributed to the correction of histological changes, oxidative stress and acetylcholinesterase activity induced by scopolamine and significantly reduced β-amyloid deposition [9].

An aqueous extract of *S. officinalis* L. has been found to exhibit insulin-like activities, while its methanolic extracts significantly decreased serum glucose in type I diabetic rats without affecting pancreatic insulin production [10]. Methanol extracts of *S. santolinifolia* Boiss. and *S. officinalis* L. showed the highest α-amylase inhibition percentage, almost comparable to that of acarbose generic anti-diabetic drug, used to treat type 2 diabetes mellitus and, in some countries, prediabetes [11].

Some diterpenoids isolated from the roots of *S. officinalis* L. have been found to have cytotoxic and DNA-damaging activity in human colon carcinoma Caco-2 cells and human hepatoma HepG2 cells in vitro [12]. Essential oil of *S. officinalis* L. and its three principal components (α-thujone, 1,8-cineole and camphor) induced a significant reduction in cell viability in LNCaP (prostate carcinoma), MCF7 (breast adenocarcinoma) and HeLa (cervical carcinoma) cell lines after 48 h of incubation [13]. Trans-caryophyllene, the main component of the sesquiterpene fraction in *S. officinalis* L., demonstrated high cytotoxic activity on amelanotic melanoma C32 and renal cell adenocarcinoma ACHN [14].

The most common polysaccharides in *S. officinalis* L. are arabinose, galactose, glucose, mannose, xylose, uronic acids and rhamnose, which are thought to be associated with immunomodulatory activity [15].

Comparing the phytochemicals in flowers, leaves, and stems of *S. officinalis* L., linalool is the most common in stems; flowers show the highest percentage of α-pinene and cineole, while bornyl acetate, camphene, camphor, humulene, limonene, and thujone are the most common in the leaves. However, it should be considered that the chemical composition of the plant would be varied depending on the environmental conditions, such as climate, water availability, and altitude [4].

It has also been shown that extracts and essential oils obtained from sage exhibit anti-inflammatory, chemoprotective, hepatoprotective, antibacterial and antiviral properties, including anti-HIV [16,17]. Antimutagenic, hypoglycemic, hypolipidemic, and analgesic properties of sage extracts are also known [18]. In traditional Chinese medicine, *S. miltiorrhizae* Radix et Rhizoma (Danshen) has been used for more than 2000 years to promote blood circulation and relieve pain [19]. Chronic hepatitis and liver fibrosis have also been treated with Danshen for centuries. The alcohol extract of Danshen is particularly rich in abietanoids and diterpene quinone pigments [20]. Currently, drugs obtained mainly from cultivated plants of *S. officinalis* L. are used in the industry [21].

Overall, more than 1000 species of sage are known in the world, including eight in Kazakhstan, of which we have chosen two [22].

The desert sage (*S. deserta* Schang.) is a valuable melliferous plant, 45–80 cm in height, with straight stems that are branched in the upper part, longer than the inflorescence, and densely pubescent; the plant grows in sandy meadows and along streams in Altai, Caucasus, south of Western Siberia, China (Xinjiang), Kazakhstan and Kyrgyzstan at an altitude of 300 to 1800 m. In traditional medicine, it is used to treat cough and urethritis [23,24].

The diterpenoid quinones 6,7-dehydroxyleanone and 6,7-dehydroroyleanone have effects in preventing myocardial ischemia, inhibiting platelet aggregation and inducing nitric oxide synthase in vitro, and the triterpenoid oleanolic acid can significantly inhibit collagen and adenosine diphosphate-induced platelet aggregation, equivalent to aspirin, to protect the heart. Extracts of *S. deserta* Schang. roots, rich in horminone, 7-O-acetylhormione and 6,7-dehydeoroyleanone, significantly inhibited FeCl_3_-induced rat carotid artery thrombosis [23]. Salvidesertones E and F, horminone, taxoquinone, 7α-O-methylhorminone, and 8α,9α-epoxy-6-deoxycoleon U showed more potent antiproliferative effects against lung adenocarcinoma cell line A549 than the positive control cisplatin [25]. Several diterpenoids have been reported to possess antiplasmodial activity [26].

The clary sage (*S. sclarea* L.) is a shrub with a height of 100–120 cm (its cultivated form may reach up to 2 m). Italy is considered the birthplace of this honey varietal (with productivity of 170 kg/ha). It is found naturally in Central and Southern Europe, Western and Central Asia, and in the Caucasus. It grows on stony, clayey slopes.

Traditionally, clary sage oil was used as an agent against gingivitis, stomatitis and aphthae. Apart from that, recent studies have reported its analgesic, anti-inflammatory, antimicrobial, antidiabetic and cytotoxic effects [27].

α-linolenic acid is the main fatty acid of the sage seed, while β-pinene and limonene are dominant volatile compounds [28].

Sclareol, another part of its essential oil, exhibits anti-inflammatory and antioxidant activity, and has an analgesic effect [29], while linalool and linalyl acetate inhibit the growth of the pathogenic fungi *Rhizoctonia solani*, *Botrytis cinerea*, *Fusarium oxysporum*, and *Alternaria solani* at concentrations of 800, 1600, 3200 and 3200 ppm [30].

Clary sage oil is also an effective bacterial inhibitor and bactericide, with a broad antibacterial spectrum against gram-positive bacteria (*Staphylococcus aureus*, *Bacillus subtilis*) and gram-negative bacteria (*Escherichia coli*, *Salmonella typhimurium*, *Klebsiella pneumonia*, *Pseudomonas aeruginosa*, *Bacillus pumilus*) [31].

Other active ingredients, contained in the inflorescence and leaves, are camphor, sabinol, α-thujone and 1,8-cineole, which exhibit mainly antimicrobial and antibiotic properties, while fatty acids (palmitic, palmitoleic, stearic, oleic, linoleic, linolenic, and arachidic) from seed extracts are thought to be responsible for its antioxidant effect [32].

The aim of the study was to evaluate the immunomodulatory effects of plant extracts from *S. deserta* Schang. and *S. sclarea* L. obtained from the plants growing in southern Kazakhstan by conventional and ultrasonic-assisted extraction.

## 2. Results

### 2.1. Testing the Quality of Raw Materials

To obtain quality products, it is important to use quality material. In addition to general identification and visual evaluation, assessment of numerical indicators is significant. In particular, ash, insoluble in 10% HCl, is represented mainly by SiO_2_ and characterizes the contamination of raw materials with mineral impurities. The studied plants comply with the basic requirements for medicinal plant raw material by the State Pharmacopeia of the Republic of Kazakhstan (SP RK) [3,33,34,35], Table 1.

The numerical indicators of plant quality, as well as the indicators of their microbiological purity and radionuclide control (established commercially at the Testing Center of JSC “Scientific Center for Anti-Infectious Drugs”), testify to their quality and, consequently, to the possibility of getting valuable extracts on their basis.

When evaluating and standardizing medicinal plant raw material, wherever possible, quantitative analysis of biologically active and/or extractive compounds is also included [3,33,34,35]. The phytochemical analysis (Table 2) showed the presence of hydrophobic compounds, amino acids, polysaccharides, and a significant number of phenolic compounds, particularly flavonoids, tannins and phenolic acids.

### 2.2. Preparation of the Extracts

The choice of the optimal technology for isolation of an extract from a studied plant material included a number of stages and provided for its most complete depletion in order to maximize the efficiency of obtaining the desired complex of valuable constituents. For this, the selection of the most suitable concentration of the extragent, its optimal ratio with the raw material, as well as the duration, temperature and frequency of extraction were elaborated. Two methods were used for isolation of extracts: conventional extraction and ultrasonic-assisted extraction.

**Figure 1:**
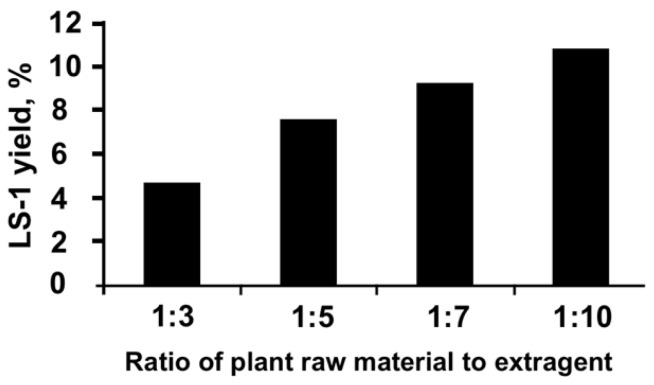
Elaborating optimal technology for LS-1: choosing the correct ratio of raw material to extragent.

It was found that the maximum yield of extract LS-1 from *S. deserta* Schang. by the method of conventional extraction is achieved when it is extracted with 50% ethanol, with the ratio of the selected extragent to raw material equal to 1:10 (Figure 1).

**Figure 2:**
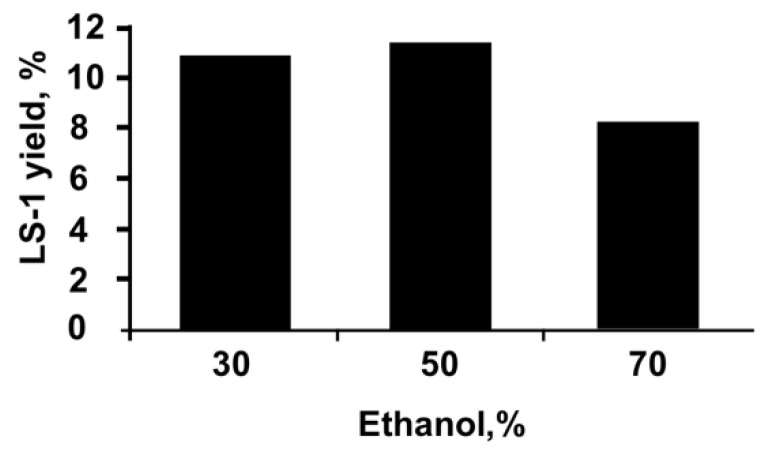
Elaborating optimal technology for LS-1: choosing correct concentration of extragent.

To find the best concentration of the extragent for obtaining the LS-1 extract, 50 g of selected plant raw material was extracted using aqueous solutions of 30%, 50% and 70% ethanol as extragent at a temperature of 30 °C for 60 min (Figure 2).

As can be seen from the averaged data presented on Figure 2, a higher yield of LS-1 extract is achieved when the raw material is extracted with 50% ethanol. The same is true for LS-3.

**Figure 3:**
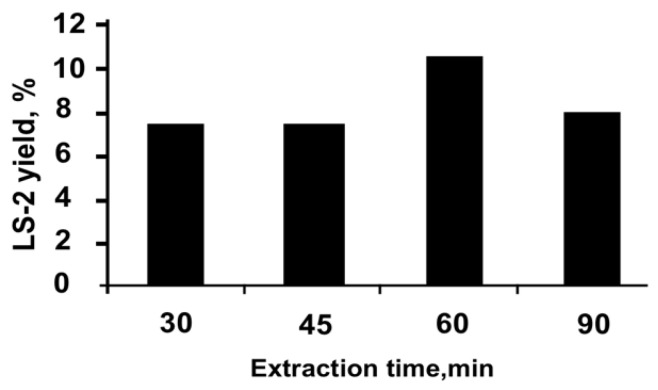
Elaborating optimal technology: choosing extraction time for LS-2.

To establish the optimal time for ultrasonic-assisted extraction, the duration of the extraction process was changed from 30 to 90 min at an optimal temperature of 30 °C, with the ratio of raw material to extragent equal to 1:10 (Figure 3).

As can be seen from the data presented on Figure 3, the maximum yield of the extract LS-2 is reached within 60 min. Thus, by varying the parameters of ultrasonic-assisted extraction in triplicate, optimal conditions for obtaining the extract LS-2 were elaborated: extraction of raw material with 50% ethanol at a ratio of raw material to extragent equal to 1:10 at a temperature of 30 °C for 60 min. The same conditions were proven to work well for obtaining the extract LS-4.

The resulting extracts are crystalline with a bitter, astringent taste, with a weak specific odor, formed of anisodiametric crystals in the form of plates, with complex ununiformed surfaces. They are insoluble in hydrophobic solvents and soluble in hydrophilic solvents—dimethyl sulfoxide, dimethylformamide, in aqueous solutions of ethanol and acetone (30% and 50%), and moderately soluble in water at 25 °C. Like all plant extracts, they are characterized by low flowability due to their strong hygroscopicity.

The results of a comparative analysis of the quantitative content of main BAC groups in extracts LS-1 and LS-2, as well as LS-3 and LS-4 are presented in Table 3 and Table 4.

### 2.3. Chemical Profiling of LS-2 and LS-4 Extracts Using LC-DAD-QTOF-MS

**Figure 4:**
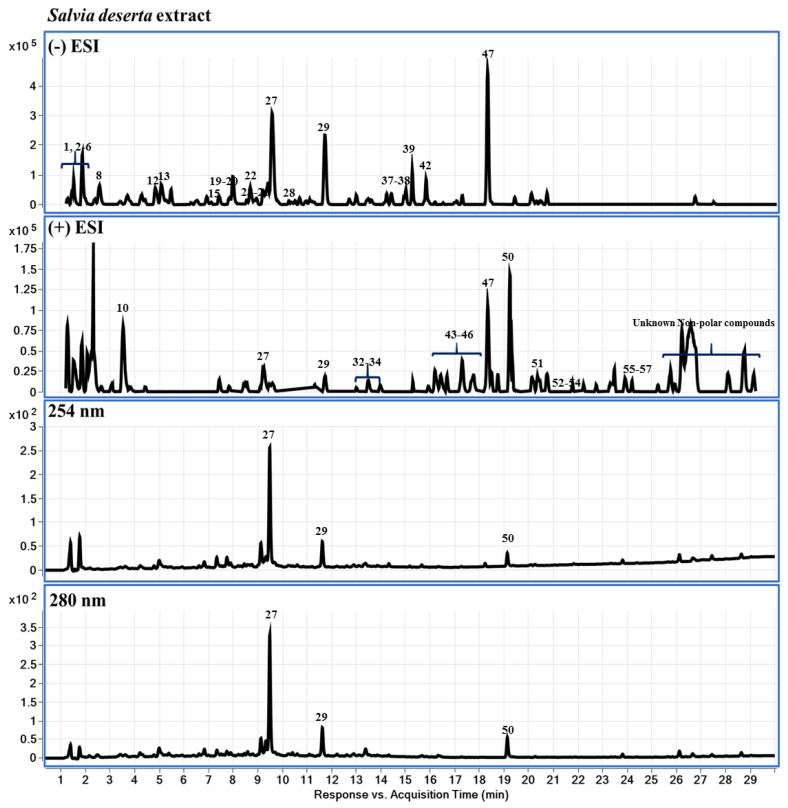

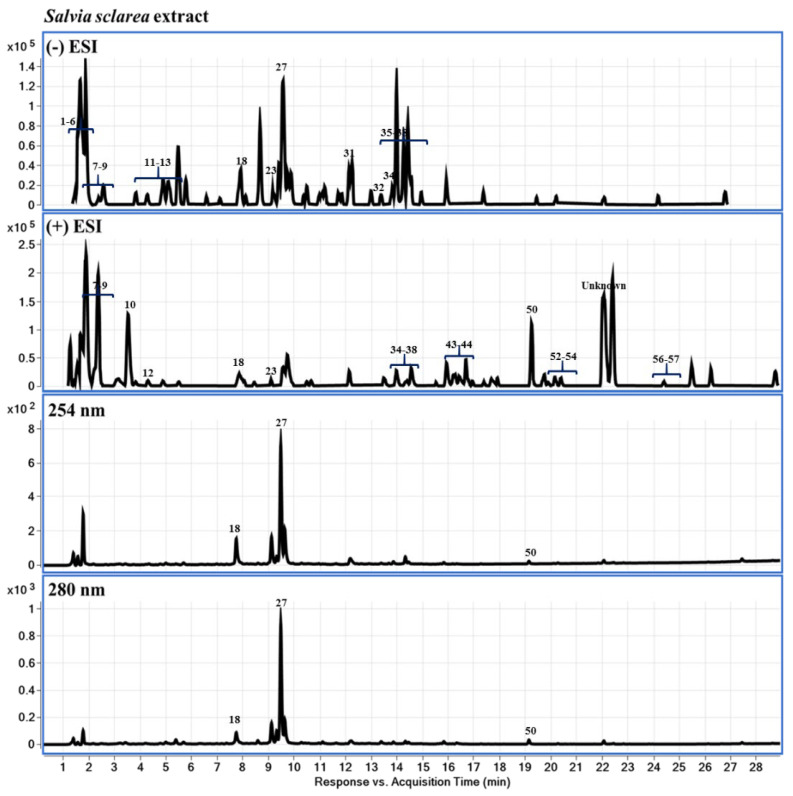
Base peak chromatograms (BPC) from *S. deserta* Schang. and *S. sclarea* L. extracts LS-2 and LS-4 in ESI positive ion mode. Each sample was injected twice.

Extracts LS-2, LS-4 obtained by ultrasonic-assisted extraction from *S. deserta* Schang. and *S. sclarea* L., respectively, were further studied using LC-DAD-QTOF-MS (or liquid chromatography-diode-array detector-quadrupole time-of-flight mass spectrometry), with the results presented in Figure 4 and described below. The corresponding data are presented in Table A1, Appendix A.

According to our results, the following compounds were detected: characteristic both for LS-2 and LS-4—valine, leucine/isoleucine, tyrosine, succinic acid, phenylalanine, danshensu (danshensuan A, 3,4-dihydroxy-phenyllactic acid), vanillic acid, tryptophan, dihydrocaffeic acid or DHCA, protocatechualdehyde/monohydroxy benzoic acid (HBA), caffeic acid, luteolin 7-O-glucoside, luteolin 7-O-glucuronide, apigenin-7-O-glucoside (flavone glycosides), apigenin 7-O-glucuronide, salvianolic acid K, chrysoeriol 7-glucuronide, rosmarinic acid, lithospermic acid B, tetramethoxyflavone, luteolin, methoxycoumarin, apigenin (flavone), jaceosidin (trihydroxyflavone), viscosine, cirsimaritin, eupatorin (3′,5-dihydroxy-4′,6,7-trimethoxyflavone), genkwanin (flavone), salvigenin (5-hydroxy-4′, 6, 7-trimethoxy flavone), maslinic acid, hydroxyursolic acid, 2α-hydroxyursolic acid, oleanolic acid, 3-epiursolic acid, of which some are unique to the genus. In addition, the following compounds were detected: specifically in LS-2—ursolic acid, 1,11,20-trihydroxy-3-lupanone (1β,11α)-form, tormentic acid, labdadien-olides, carnosic acid, hydroxycarnosic acid, rosmanol, salvianolic acid B, horminone, apigenin-rutinoside isomer, isoquercitrin (quercetin 3-O-β-D-glucopyranoside), ferulic acid, luteolin 7-O-β-rutinoside, quercetin glycoside; in LS-4—salviaflaside, kaempferol-glucoronide, hispidulin, 3′,4′,5-trihydroxy-7-methoxyflavone, dihydroxy-dimethoxyflavone, indicating that some species-specific compounds were also found.

### 2.4. Cell Viability

**Figure 5:**
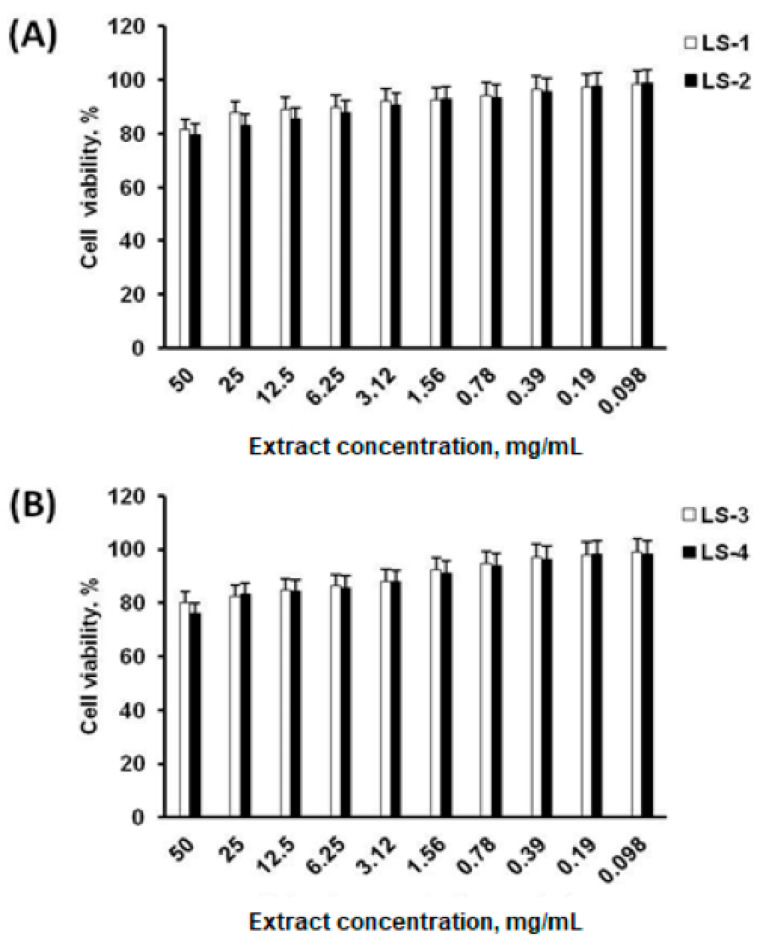
Effects of *S. deserta* Schang. (**A**) and *S. sclarea* L. (**B**) extracts on cell viability of neonatal human dermal fibroblasts. (LS-1, LS-3) sage extracts obtained by conventional extraction and (LS-2, LS-4) ultrasonic-assisted extraction.

Cytotoxic effects of sage extracts of *S. deserta* Schang. (LS-1 and LS-2) and *S. sclarea* L. (LS-3 and LS-4), obtained by conventional extraction and ultrasonic-assisted extraction, on neonatal human dermal fibroblasts (HDFn) were evaluated using the 3-(4,5-dimethylthiazol-2-yl)-2,5-diphenyl-2H-tetrazolium bromide (MTT) assay. The results of the MTT assay (Figure 5) showed that all the obtained sage extracts from *S. deserta* Schang. (Figure 5A) and *S. sclarea* L. (Figure 5B) do not affect the cell viability of HDFn at different concentrations.

Cell viability after incubation with sage extracts at a concentration of 50 mg/mL was more than 70% with median cellular cytotoxic concentrations (CC50) values > 50 mg/mL, officially accepted, which indicates that even at a higher concentration, sage extracts do not cause a significant toxic effect on human fibroblasts. In addition, notable differences between sage extracts obtained by conventional (LS-1, LS-3) and ultrasonic-assisted extraction (LS-2, LS-4) were not found.

### 2.5. Immunomodulatory Effects of Sage Extracts

In this study we compared immunomodulatory effects of the extracts of *S. deserta* Schang. (LS-1 and LS-2) and *S. sclarea* L. (LS-3 and LS-4) obtained by conventional and ultrasonic-assisted extraction on pro-inflammatory cytokine secretion and phagocytic activity of murine immune cells.

To compare the immunomodulatory activity of sage extracts obtained by two different extraction methods, commercial ELISA kits were used to determine the levels of proinflammatory cytokine secretion (TNF-α and IL-1β) by murine peritoneal macrophages and spleen T lymphocytes. Two controls were used in the experiment: macrophages/T-lymphocytes without treatment and macrophages/T lymphocytes activated by lipopolysaccharide (LPS) or concanavalin A (ConA), respectively.

**Figure 6:**
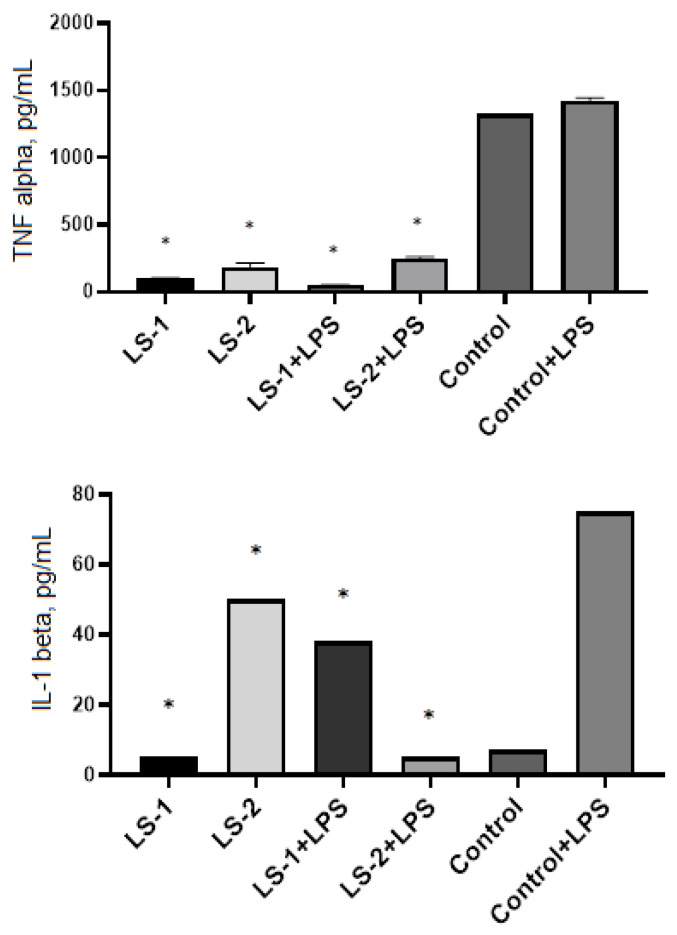
Effects of *S. deserta* Schang extracts on the secretion of TNF-α and IL-1β by murine peritoneal macrophages. (LS-1)—sage extract obtained by conventional extraction and (LS-2)—sage extract obtained by ultrasonic-assisted extraction. Two controls were used in the experiment: macrophages without treatment or stimulus and macrophages activated with LPS. (*) indicates significant differences in comparison to control, *p* < 0.05.

In the wells with macrophages and T lymphocytes, 50 mg/mL of plant extracts were added and incubated for 24 h at 37% and 5% CO_2_. ELISA showed that extracts of *S. deserta* Schang. (LS-1 and LS-2) significantly suppressed the level of TNF-α production in both intact and LPS-activated macrophages (Figure 6). At the same time, no significant difference was found between the effects of extracts LS-1 (conventional extraction) and LS-2 (ultrasonic-assisted extraction).

Measurement of IL-1β levels in intact macrophages showed that sage extract obtained by ultrasonic-assisted extraction (LS-2) caused a significant increase in IL-1β level compared to the control and LS-1 sage extract. In contrast, the level of IL-1β production in LPS-activated macrophages was significantly suppressed after incubation with LS-2 sage extract.

**Figure 7:**
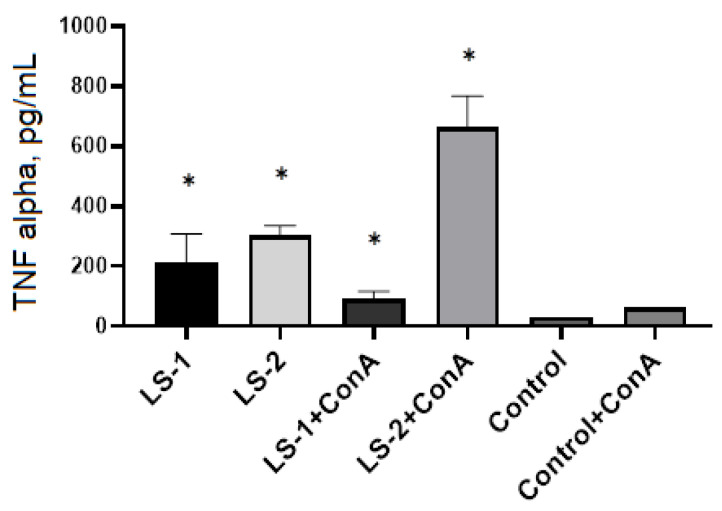
Effects of *S. deserta* Schang. extracts on the secretion of TNF-α by spleen T lymphocytes. (LS-1)—sage extract obtained by conventional extraction and (LS-2)—sage extract obtained by ultrasonic-assisted extraction. Two controls were used in the experiment: spleen T lymphocytes without treatment or stimulus and spleen T lymphocytes activated with Con A. (*) indicates significant differences in comparison to control, *p* < 0.05.

The study of the effect of sage extracts of *S. deserta* Schang. on TNF-α production by spleen T lymphocytes showed that sage extracts induced a significant increase in TNF-producing activity in both intact and Con A—activated T lymphocytes (Figure 7). The greatest increase in TNF-α secretion was found after treatment of spleen T lymphocytes with sage extract (LS-2) obtained by ultrasonic-assisted extraction.

**Figure 8:**
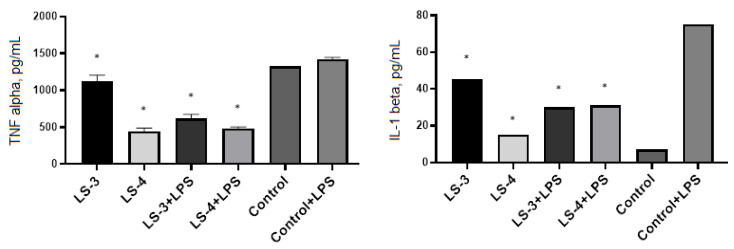
Effects of *S. sclarea* L. extracts on the secretion of TNF-α and IL-1β by murine peritoneal macrophages. (LS-3)—sage extract obtained by conventional extraction and (LS-4)—sage extract obtained by ultrasonic-assisted extraction. Two controls were used in the experiment: macrophages without treatment or stimulus and macrophages activated with LPS. (*) indicates significant differences in comparison to control, *p* < 0.05.

The same ELISA study was conducted for extracts from *S. sclarea* L. Comparative analysis of the immunomodulatory activity of *S. sclarea* L. extracts obtained by conventional extraction (LS-3) and ultrasonic-assisted extraction (LS-4) showed that both sage extracts significantly suppressed the level of TNF-α production in both intact and LPS-activated macrophages (Figure 8).

ELISA has revealed that the treatment of macrophages with *S. sclarea* L. extracts has a different effect on the level of IL-1β production by macrophages, for instance, with secretion of IL-1β in the intact macrophages after treatment with *S. sclarea* L. extracts, especially LS-3. On the other hand, *S. sclarea* L. extracts suppress the production of this pro-inflammatory cytokine by LPS-activated macrophages.

**Figure 9:**
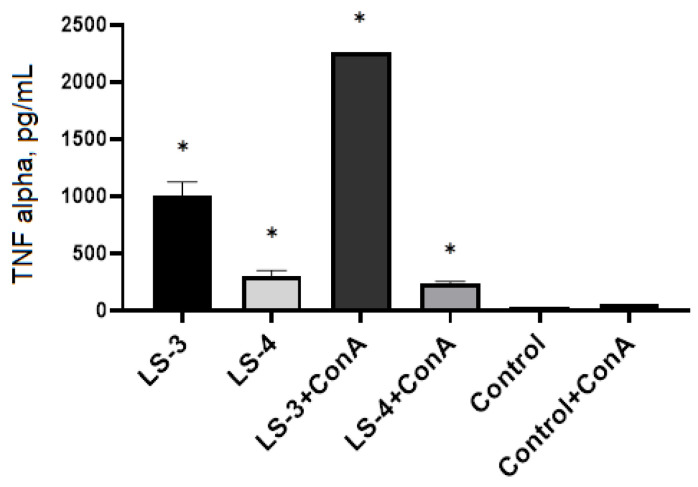
Effects *S. sclarea* L. extracts on the secretion of TNF-α by spleen T lymphocytes. (LS-3)—sage extract obtained by conventional extraction and (LS-4)—sage extract obtained by ultrasonic-assisted extraction. Two controls were used in the experiment: spleen T lymphocytes without treatment or stimulus and spleen T lymphocytes activated with Con A. (*) indicates significant differences in comparison to control, *p* < 0.05.

The analysis of TNF-producing activity of spleen T lymphocytes demonstrated that *S. sclarea* L. extracts resulted in increase in the level of the production in both intact and Con A-activated T lymphocytes (Figure 9).

Besides analysis of the cytokine-producing activity of immune cells, the phagocytic activity of peritoneal macrophages has been also studied after treatment with extracts of *S. deserta* Schang. and *S. sclarea* L.

**Figure 10:**
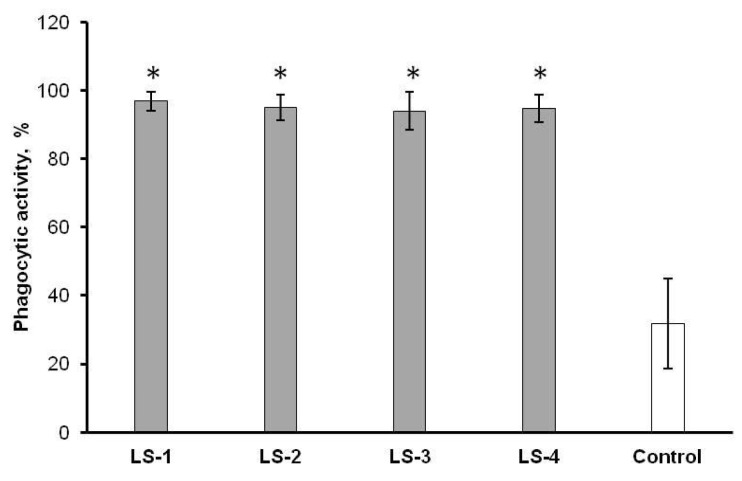
Effects of *S. deserta* Schang. (LS-1, LS-2) and *S. sclarea* L. (LS-3, LS-4) extracts on the phagocytic activity of peritoneal macrophages. (LS-1, LS-3) sage extracts obtained by conventional extraction and (LS-2, LS-4) sage extracts obtained by ultrasonic-assisted extraction. (*) indicates significant differences in comparison to control, *p* < 0.05.

The results of a comparative analysis of the effects of sage extracts showed that, regardless of the type of sage extract and the extraction method, all sage extracts significantly increase the phagocytic activity of peritoneal macrophages (Figure 10).

There was no significant difference in macrophage phagocytic activity between the studied extracts.

## 3. Discussion

It is well known that medicinal plants are a source of a large number of biologically active compounds (polyphenols, flavonoids, polysaccharides, alkaloids, saponins, terpenes, lipids, etc.) capable of modulating the cellular and humoral immune responses [36,37]. Immunomodulatory potential of such can be used for treatment of immune-mediated inflammatory diseases, infections and tumors [38,39].

At present, there is a number of studies on the immunomodulatory effects of different *Salvia* species. As such, it has been reported that the extracts of *S. miltiorrhiza* significantly enhanced cell-mediated immunity in healthy subjects and breast cancer patients [40,41]. Moon et al. have demonstrated that extracts of *S. miltiorrhiza* inhibited the production of pro-inflammatory cytokines via down-regulation of gene and protein expression, whereas it increased the anti-inflammatory cytokines in LPS-induced macrophages [42]. Other researchers revealed that different tanshinones from *S. miltiorrhiza* markedly inhibited production of IL-12 in activated macrophages and antigen-presenting cells [43]. Shin et al. showed immunostimulatory effects of *S. plebeia* R. aqueous extract significantly increased phagocytic activities, nitric oxide (NO) production, and pro-inflammatory cytokines TNF-α and IL-1β in Raw264.7 macrophages [44].

In our study, we examined the effects of the extracts of *S. deserta* Schang. and *S. sclarea* L. obtained by conventional and ultrasonic-assisted extraction on the production of pro-inflammatory cytokines and phagocytic activity of murine immune cells. Before the investigation of the immunomodulatory effects of sage extracts, we first evaluated the cytotoxicity of different concentration of the extracts of *S. deserta* Schang. and *S. sclarea* L. (0.098∼50 mg/mL) in dermal fibroblasts. The results of the cytotoxicity assay indicated that the tested concentrations of sage extracts did not affect cell viability.

In order to study the immunomodulatory effects of sage extracts, we conducted ELISA for measurement of TNF-α and IL-1β in murine peritoneal macrophages and splenic T lymphocytes with and without stimulation by LPS or Con A. LPS and Con A were used as immunogenic stimulators to produce cytokines by macrophages and T lymphocytes, respectively. TNF-α and IL-1β are mainly produced by macrophages and monocytes and are considered the most important cytokines that promote inflammatory responses [45].

Our results showed that the extracts of *S. deserta* Schang. and *S. sclarea* L. obtained by conventional and ultrasonic-assisted extraction significantly reduced secretion of pro-inflammatory cytokine TNF-α in both unstimulated and LPS-stimulated peritoneal macrophages. However, interesting data have been obtained when measuring IL-1β levels in macrophages. When unstimulated macrophages were treated with *S. deserta* Schang. extract (LS-2) and *S. sclarea* L. extract (LS-3) the release of IL-1β in the macrophages was significantly increased in comparison to control. On the other hand, when LPS-stimulated macrophages were treated with the same sage extracts, the level of IL-1β secretion in macrophages was significantly inhibited.

The data obtained by us are in good agreement with the data obtained by other scientists, who showed that several groups of plant secondary metabolites impact the functional activity of macrophages, including phenolic compounds, terpenes, and polysaccharides [46]. For instance, it has been reported that the extract of *S. miltiorrhiza* inhibits the production of nitric oxide, PGE2, IL-1β, IL-6, and TNF-α, and the expression of chemokines, RANTES, CX3CL1, CX3CR1, as well as inflammatory mediators such as TLR-4 and 11β-HSD1 in LPS-stimulated RAW 264.7 macrophages [43]. On the contrary, Im et al. found that polysaccharides isolated from *Salicornia herbacea* stimulate intact murine macrophages to release high levels of cytokines TNF-α, IL-1β and nitric oxide [47]. Other researchers demonstrated that pretreatment with *S. miltiorrhiza* polysaccharides lead to a significant reduction of TNF-α, IL-6 and IL-1β in LPS-stimulated macrophages [48].

Several studies have shown that plant polysaccharides can regulate phagocytosis of macrophages [49,50,51]. Phagocytosis is an essential element for host defense which combines bacterial elimination with initiation of the innate immune response and antigen presentation to T lymphocytes. In our study we revealed that treatment of intact peritoneal macrophages with examined plant extracts significantly increased the phagocytic activity of macrophages in comparison to a control. We suggest that the immunostimulatory effects observed in our study are associated with sage polysaccharides, which may bind to mannose receptors on macrophages and activate their phagocytic activity, production of reactive oxygen species and the secretion of cytokines.

T lymphocytes are the major cell population of the adaptive immune system. They play key roles in elimination of infected and foreign cells, activation of other immune cells, secretion of cytokines and modulation of the immune response [52]. Recently, Chen et al. demonstrated that *S. miltiorrhiza* polysaccharide can specifically promote the proliferation and enhance cytotoxicity of T lymphocytes in peripheral blood of cancer patients through the activation of TLRs, MAPK and NF-κB signaling pathways [53]. In our study, we have also found that extracts of *S. deserta* Schang. (LS-2) and *S. sclarea* L. (LS-3) significantly increased secretion of TNF-α by intact and Con A—activated splenic T lymphocytes. The greatest immunostimulant effect was found in T lymphocytes treated with sage extracts LS-2 and LS-3.

Thus, based on our results and literature data, we suggest that extracts of *S. deserta* Schang. (LS-2) and *S. sclarea* L. (LS-3) contain polysaccharides with remarkable biological properties able to modulate the functional activity of macrophages and T lymphocytes depending on their physiological state, and might be used for further preclinical and clinical studies.

## 4. Materials and Methods

### 4.1. Checking the Quality of Plant Materials and Obtaining the Extracts

**Figure 11:**
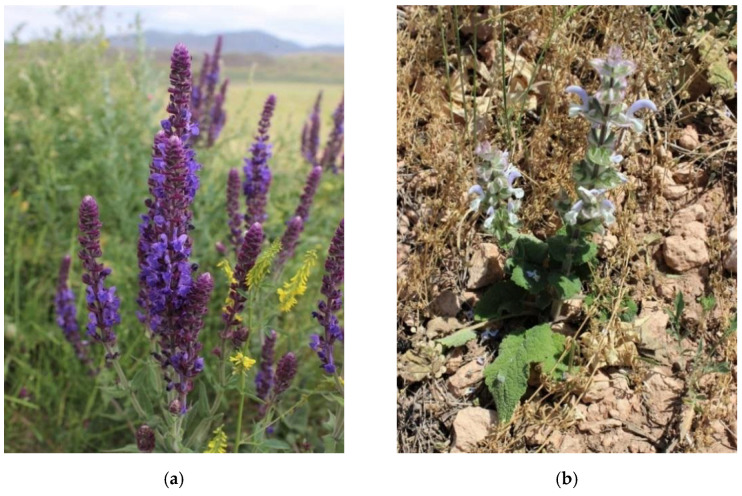
Appearance of harvested plants: (**a**)—*S. deserta* Schang., (**b**)—*S. sclarea* L.

The harvesting of two investigated species of plants of the genus sage was carried out during their flowering period, June 2021. During the trip to the territory of the Kordai district of the Zhambyl region (the total length of the route: Almaty-Otar-Kenen-UlkenSulutor-Kenen-Otar-Almaty was 400 km) the coordinates of the place of collection and growth of the species *S. sclarea* L., *S. deserta* Schang. are marked (Table 5), photos of the objects of the study were taken (Figure 11), and raw material was collected for study and production of extracts.

After the collection and initial treatment of plant materials (cleaning, drying and crushing) numerical indicators were determined to test their quality using the methods of SP RK in triplicate, with average results presented in Table 1 [33,34,35]. In addition, indicators of their microbiological purity and radionuclide have been tested commercially in a certified laboratory of the of JSC “Scientific Center for Anti-Infectious Drugs” Testing Center.

### 4.2. Preparation of Extracts

To elaborate the optimal conditions for obtaining the extracts, 30%, 50%, and 70% ethanol solutions were used with the choice of the most suitable concentration, providing a more complete depletion of raw materials; the ratio of the selected extragent to raw material was established with a change in their ratio in the generally accepted range for the aerial part of medicinal plants from 1:5 to 1:10, the extraction time (with a change in it in the range from 5 to 48 h) and the extraction rate (when its number changed from one to three). Dry extracts were obtained using a rotary evaporator model IKA RV20 (IKA^®^-Werke GmbH & Co. KG, Staufen, (Germany). The mode of ultrasonic waves was changed in the range of 30–40 kHz, the power 300–1200 W, the time of ultrasound exposure was 10–60 min, and the temperature was 30–60 °C [54,55]. The parameter of the optimization of the extraction process was the yield of extracts and the quantitative content of tannins and polysaccharides in it.

For chromatographic control of the studied objects, we used the methods of one-dimensional and two-dimensional chromatography on paper and thin layer chromatography using standard samples, specific developers, diagnosing and complexing reagents. Chromatography on FN3 paper (Sartorius Lab Instruments GmbH & Co. KG, Goettingen, Germany) was carried out in solvent systems (ratio by volume): n-butanol-acetic acid-water (40:12.5:29); n-butanol-acetic acid-water (4:1:5); benzene-acetic acid-water (6:7:3); n- butanol -pyridine-water (6:4:3); acetic acid (2–15%); in a thin layer on TLC plates Silica gel F254 (Merck KGaA, Darmstadt, Germany) in solvent systems (ratio by volume): hexane-ethyl acetate (1:1; 2:1; 3:1; 7:3), chloroform-methanol-water (95 5:0.5; 80:20:2; 85:15:1.5); chloroform-methanol (95:5; 9:1; 85:15; 8:2); butanol-acetic acid-water (4:1:5; 10:1:3; 40:12.5:29); butanol-acetone-water (3:1:5); chloroform-ethanol (8:2), developed by cerium sulfate, ammonia vapor, 1% vanillin solution in HCl, 1% solutions of aluminum chloride, iron ammonium alum, o-toluidine and ninhydrin developers, diazotized p-nitroaniline, diazotized sulfanilic acid, 10% H_2_SO_4_.

All high-purity chemicals were obtained from Sigma-Aldrich (St. Louis, MA, USA). All solutions were prepared with ddH_2_O.

The quantitative content of tannins in extracts was determined spectrophotometrically using a standard sample [56] on a JASCO J-715 spectrophotometer (Jasco Corp., Tokyo, Japan). The quantitative content of polysaccharides was determined by the pharmacopeial method [33,34,35].

### 4.3. Chemical Profiling of LS-2 and LS-4 Extracts Using LC-DAD-QTOF-MS

Extracts LS-2 and LS-4 from *S. deserta* Schang. and *S. sclarea* L., obtained by ultrasonic-assisted extraction, were concentrated to dryness using a rotary evaporator RV 10 digital V (IKA, Germany) under a temperature of 40–45 °C and further used for analysis. The liquid chromatographic system was an Agilent Series 1290, and the mass spectrometric analysis was performed with a QToF-MS/MS (Model #G6530A, Agilent Technologies, Santa Clara, CA, USA) equipped with an ESI source. All operations, acquisition and analysis of data were controlled by Agilent MassHunter Acquisition Software vA.01.00 (Agilent Technologies, Santa Clara, CA, USA) and operated under MassHunter Workstation software vB.02.00 (Agilent Technologies, Santa Clara, CA, USA). Each sample was analyzed in positive and negative modes to provide abundant information for structural identification [57]. Mass spectra were recorded across the range *m*/*z* = 50–1300 with accurate mass measurement of all mass peaks (Figure 4). MassHunter Workstation software, including Qualitative Analysis (vB.07.00), was used for processing both raw MS and MS-MS data, including molecular feature extraction, background subtraction, data filtering, and molecular formula estimation. The raw data were processed using the Find by Molecular Feature (MF) algorithm called Molecular Feature Extractor (MFE) within MassHunter Qualitative Analysis software (Agilent Technologies, Santa Clara, CA, USA). All the solvents, acetonitrile, methanol, formic acid used are of HPLC-certified grade, obtained from ThermoFisher Scientific (Hampton, NH, USA). Water for the mobile phase was purified using a Milli-Q system (Millipore, Burlington, MA, USA). Extracted molecular features were processed to create a list of compounds.

### 4.4. Animals

Male C57BL/6 mice 10–12-weeks old were purchased from SPF-vivarium of Masgut Aikimbayev’s National Scientific Center for Especially Dangerous Infections (Almaty, Kazakhstan). The animals were housed in a temperature-controlled environment (23 °C) with 60% relative humidity applying a 12 h light/dark cycle. The animals had ad libitum access to food and water. Experimental procedures involving animals were in full compliance with current international laws and policies [58] and were approved by the Local Ethics Committee for Animal Use in the National Center for Biotechnology (Kazakhstan).

### 4.5. Cell Line

Neonatal human dermal fibroblasts (HDFn) employed in this study were obtained from Gibco (ThermoFisher Scientific, Carlsbad, CA, USA). HDFn were cultured in complete nutrient medium Dulbecco’s Modified Eagle Medium (DMEM) containing 4.5 g/L glucose, 10% fetal bovine serum (FBS), 1% penicillin-streptomycin at 37 °C and 5% CO_2_. The adherent cells were passed with TrypLE^®^ Express (ThermoFisher Scientific, Paisley, UK) with an interval of 5–6 days. The medium in the cell culture was changed every two days. The cells were counted using a BioRad TC-20 automated cell counter (BioRad Laboratories, Hercules, CA, USA).

### 4.6. Isolation of Peritoneal Macrophages

Murine peritoneal macrophages were isolated according to a standard protocol described previously by Zhang et al. [59]. The mice were sacrificed by cervical dislocation and peritoneal macrophages were isolated by lavages of the peritoneal cavity with cold basal culture medium DMEM (Gibco, USA). The resident macrophages were collected by aspiration of the lavage fluid into a 10-mL syringe and harvested by centrifugation for 10 min at 400× *g* and 4 °C. The cells were resuspended in cold complete culture medium DMEM supplemented with 10% FBS (Gibco, USA) and 1% penicillin–streptomycin (Gibco, USA). The viability and number of peritoneal cells was determined by trypan-blue dye exclusion using a BioRad TC-20 automated cell counter (BioRad, Rocklin, CA, USA). In order to separate macrophages from other peritoneal cells, 2 × 10^6^ nucleated cells were added to 6-well tissue culture plates and allowed to adhere to the substrate by culturing them for 1 h at 37 °C in CO_2_-incubator. Nonadherent cells were removed by gently washing three times with warm DMEM. After separation on cell adhesion, peritoneal macrophages were greater than 90%.

### 4.7. Isolation of Spleen Lymphocytes

Murine lymphocytes were isolated from the spleen according to a standard protocol described previously by Lim et al. [60]. For isolation of the spleen lymphocytes, animals were sacrificed by cervical dislocation. Spleens were removed aseptically and ground in glass homogenizers in cold RPMI-1640 medium (Sigma, USA) containing 2% FBS, penicillin (100 IU/mL)/streptomycin (100 µg/mL) and 55 µM 2-mercaptoethanol. Red blood cells (RBC) were lysed by treatment with RBC lysis buffer; cells were washed twice with phosphate buffered saline (PBS). Then, the cells were resuspended in RPMI-1640 medium supplemented with 5% FBS and penicillin (100 IU/mL)/streptomycin (100 µg/mL) to a final concentration of 7 × 10^6^ cell/mL. Splenic B and T lymphocyte populations were fractionated by panning [61]. Plastic cell culture flasks were coated with the affinity-purified rabbit immunoglobulin (Ig) (Sigma, USA) with specificity for mouse Ig at a concentration of 200 µg/mL in 7 mL of PBS. After incubation overnight at 4 °C, the flasks were washed thrice with PBS and left for 45 min with RPMI 1640 medium. Spleen cells were then placed into the flasks in 7 to 8 mL aliquots. Flasks were incubated at 4 °C for 60 min with gentle stirring to redistribute cells every 30 min. Nonadherent cells were carefully poured off, washed once and resuspended into RPMI-1640 medium supplemented 10% FBS, penicillin (100 IU/mL)/streptomycin (100 µg/mL) and 55 µM 2-mercaptoethanol.

### 4.8. Cytotoxicity Test

HDFn were plated into the wells of a 96-well plate (BD Biosciences, Franklin Lakes, NJ, USA) at 1 × 10^4^ cells/well. The cells were incubated in a high-glucose DMEM medium containing 10% FBS overnight at 37 °C and 5% CO_2_. Then, the culture medium was carefully replaced with fresh medium in a volume of 50 μL, and 50 μL of the plant extracts (LS-1, LS-2, LS-3, LS-4) were added. DMEM medium containing 10% FBS served as a control. The cells were incubated for 48 h at 37 °C and 5% CO_2_. After 48 h of incubation, the culture medium in each well of the 96 well plate was replaced with 90 μL of fresh DMEM medium without phenol red (Gibco, USA). Next, 10 μL of 3-(4,5-dimethylthiazol-2-yl) -2,5-diphenyltetrazolium bromide (MTT) solution (5 mg/mL) was added to each well. The cells were incubated for 4 h at 5% CO_2_ and 37 °C. After incubation, the medium was carefully removed with an aspirator and formazan was dissolved by adding 100 μL of dimethyl sulfoxide (DMSO) per well. Optical density was recorded using a BioRad 680 plate spectrophotometer (BioRad Laboratories, Marnes-la-Coquette, France) at a wavelength of 580 nm. MTT test data were obtained by comparing the optical density (OD) in the experimental groups. The optical density is proportional to the number of living cells in the well. The change in OD was judged on the cytotoxic activity of the tested samples. The value of cell viability was calculated by the formula: A test/A control × 100, where A test is the optical density of the test wells; A control—the optical density of the control wells; 100—100% value.

### 4.9. Cytokine Detection in Cell Supernatants

The measurement of cytokine levels (TNF-α and IL-1β) in cell supernatants was carried out using enzyme-linked immunosorbent assay (ELISA). Peritoneal macrophages were incubated in the presence or absence of 1 μg/mL of bacterial lipopolysaccharides (LPS) (Merck, Darmstadt, Germany), and spleen lymphocytes in the presence or absence of 2.5 µg/mL with concanavalin A (ConA) (Sigma, USA). The cells were additionally stimulated with tested plant extracts (LS-1, LS-2, LS-3, LS-4) at a final concentration 50 mg/mL. ELISA was performed using the following kits: Mouse TNF alpha ELISA Kit and Mouse IL-1 beta ELISA Kit (Abcam, Cambridge, UK). In the wells of a 96-well MaxiSorb plate (Nunc, Rochester, NY, USA), 50 μL volumes of the tested samples were added to the wells of the plate. Staining was performed using antibodies labeled with streptavidin conjugated with horseradish peroxidase at a concentration of 1:250. Detection was performed using a TMB substrate Reagent Set (BD Biosciences, USA). The ELISA results were assessed visually and quantitatively using a BioRad 680 plate spectrophotometer (BioRad, France) at a wavelength of 450 nm. The data were processed using Zemfira 4.0 software (BioRad, Hercules, CA, USA).

### 4.10. Phagocytosis Assay

To determine the phagocytic activity, peritoneal macrophages were seeded into the wells of the 24-well culture plate at 5 × 10^5^ cells/well. After 1 h incubation at 37 °C, the wells were washed two times with PBS. Then, plant extracts (LS-1, LS-2, LS-3, LS-4) diluted in complete culture medium DMEM were added to the wells containing monolayer of macrophages and incubated for 24 h at 37 °C and 5% CO_2_. The culture plate was then washed 3 times with PBS and diluted overnight bacterial culture of *Escherichia coli* (1:10) was added and incubated for 30 min with macrophages. After incubation, the cells were washed 3 times with PBS and fixed with 4% paraformaldehyde solution for 20 min. To stain gram-negative bacteria, 10 μL SYTO9 (ThermoFisher Scientific, Waltham, MA, USA) was added to each well and incubated for 10 min at room temperature. The phagocytic activity of macrophages was analyzed using an inverted fluorescence microscope Axio Observer A1 (Carl Zeiss Microscopy GmbH, Jena, Germany) and Zen 2011 software (Carl Zeiss Microscopy GmbH, Jena, Germany). The obtained images were processed using the ImageJ 1.52v software (NIH, Bethesda, MD, USA).

### 4.11. Statistical Analysis

All data are presented as mean ± standard deviation. The statistical significance was calculated using one-way analysis of variance ANOVA test. *p* < 0.05 was considered statistically significant. Statistical analysis was conducted with software Statistica 6.0 (StatSoft, Tulsa, OK, USA).

## 5. Conclusions

In the study the choice of the optimal technology for isolation of sage extracts included a number of parameters (extragent, ratio of extragent to raw material, time of extraction) provided for its most complete depletion in order to maximize the efficiency of obtaining the desired complex of valuable constituents, which demonstrated that under a significant extraction time reduction, ultrasound-assisted extraction allows obtaining a comparable amount of extracts and does not significantly impact the representation of major groups of BAC. Our study has demonstrated that the extracts of *S. deserta* Schang. (LS-2) and *S. sclarea* L. (LS-3) contain bioactive compounds, in particular polysaccharides that may stimulate or suppress the functional activity of peritoneal macrophages and T lymphocytes depending on their status. These results suggest that *S. deserta* Schang. and *S. sclarea* L. may be used as natural immunomodulators for the prevention and treatment of inflammatory diseases.

## Figures and Tables

**Table 1:** Numerical indicators of the studied plant species.

Numerical Indicators, %	*S. deserta* Schang.	*S. sclarea* L.
Moisture	8.14	8.82
Total ash	9.67	10.05
Ash insoluble in 10% HCl	1.19	1.33
Sulphated ash	9.98	10.89

**Table 2:** The quantitative content of some biologically active constituents in studied plants.

Main Groups, %	*S. deserta* Schang.	*S. sclarea* L.
Polysaccharides	1.76	2.63
Flavonoids	8.72	5.56
Organic acids	0.111	0.112
Tannins	16.42	9.98

**Table 3:** Comparative quantitative content of biologically active compounds in LS-1 and LS-2 extracts from *S. deserta* Schang.

Main Groups, %	LS-1	LS-2
Polysaccharides	1.81	1.83
Flavonoids	9.79	10.14
Organic acids	2.78	2.67
Tannins	23.35	24.06

**Table 4:** Comparative quantitative content of biologically active compounds in LS-3 and LS-4 extracts from *S. sclarea* L.

Main Groups, %	LS-3	LS-4
Polysaccharides	2.67	2.68
Flavonoids	8.61	7.71
Organic acids	2.13	2.24
Tannins	9.98	10.12

**Table 5:** Coordinates of the collection of plant materials.

Species	Collection Area	GPS Coordinates	Phenophase	Collected Part	Phytomass, kg
*S. sclarea* L.	Zhambyl region, Korday region, ridge Zhetyzhol, env. pos. Ulken Sulutor	H = 1326 m above the sea level N = 43°13′368″ E = 075°10′971″	budding, beginning of flowering. sp-sol, blooming	aerial part (fresh)	26.2
*S. deserta* Schang.	Zhambyl region, Korday region, ridge Zhetyzhol, env. pos. Ulken Sulutor	H = 1339 m above the sea level N = 43°12′655″ E = 075°11′226″	budding, blooming, cop 3	aerial part (fresh)	20.0

## Data Availability

Not applicable.

## Appendix A

**Table A1:** Chemical profiling of LS-2 and LS-4 extracts using LC-DAD-QTOF-MS.

#	RT (min)	Compound Name	Mol. Formula	[M+H]+	Fragment Ions	[M-H]^−^	Fragment Ions	Identities	
								*S. deserta* Schang.	*S sclarea* L.
1	1.58	Valine	C_5_H_11_NO_2_	118.0865 (118.0863)	72.0812	-	-	+	+
2	1.6	Proline	C_5_H_9_NO_2_	116.0709 (116.0706)	70.0657	-	-	ND	+
3	1.8	Luecine/ Isoleucine	C_6_H_13_NO_2_	132.1015 (132.1019)	-	-	-	+	+
4	1.94								
5	1.85	Tyrosine	C_9_H_11_NO_3_	182.0810 (182.0812)	70.0655	-	-	+	+
6	1.99	Succinic acid	C_4_H_6_O_4_	-	-	117.0195 (117.0193)	-	+	+
7	2.4	Phenylalanine	C_9_H_11_NO_2_	166.0862 (166.0863)	120.0786, 103.0524	-	-	+	+
8	2.57	Danshensu (Danshensuan A, 3,4-dihydroxy- phenyllactic acid)	C_9_H_10_O_5_	199.0603 (199.0601)	135.0461	197.0455 (197.0455)	179.0349 [M-H-H_2_O]^−^ 135.0454 [M-H-H_2_O-CO_2_]^−^	+	+
9	2.68	Vanillic acid	C_8_H_8_O_4_	169.0493 (169.0495)	-	167.0349 (167.0350)	-	+	+
10	3.5	Tryptophan	C_11_H_12_N_2_O_2_	205.0974 (205.0972)	188.0707, 115.0543, 91.0545	-	-	+	+
11	3.8	dihydrocaffeic acid or DHCA	C_9_H_10_O_4_	-	-	181.0501 (181.0506)	161.0239; 135.0445	+	+
12	4.24	Protocatechualdehyde/Monohydroxy benzoic acid (HBA)	C_7_H_6_O_3_	139.0391 (139.039)	111.0441 [M+H-CO]^+^	137.0239 (137.0244)	109.0297 [M+H-CO]^−^	+	+
13	5.1	Caffeic acid	C_9_H_8_O_4_	181.0493 (181.0495)	163.0391 [M+H-H_2_O]^+^	179.0350 (179.035)	135.0454 [M-H-CO_2_]^−^	+	+
14	6.64	Quercetin glycoside (Flavonol glycoside)	C_21_H_20_O_12_	465.1025 (465.1028)	303.0501	463.0885 (463.0882)	300.0181	+	ND
15	7.2	Salviaflaside	C_24_H_26_O_13_	-	-	521.1298 (521.1301)	359.0715	ND	+
16	7.42	Luteolin 7-*O*-β-rutinoside	C_27_H_30_O_15_	595.1660 (595.1657)	287.0552 [M+H-162-146]^+^	593.1508 (593.1512)	285.0404 [M+H-162-146]^+^	+	ND
17	7.65	Ferulic acid	C_10_H_10_O_4_	195.0653 (195.0652)	-	193.0507 (193.0506)	149.0609 [M-H-CO_2_]^−^ 134.0370 [M-H-CO_2_-CH_3_]^−^	+	ND
18	7.83	Isoquercitrin (Quercetin 3-*O*-β-D-glucopyranoside)	C_21_H_20_O_12_	465.1029 (465.1028)	303.0490 [M+H-162]^+^	463.0881 (463.0882)	300.0188	+	ND
19	7.84	Luteolin 7-*O*-glucoside	C_21_H_20_O_11_	449.1079 (449.1078)	287.0545 [M+H-Glc]^+^	447.0931 (447.0933)	285.0399	+	+
20	7.89	Luteolin 7-*O*-glucuronide/Kaempferol-glucoronide	C_21_H_18_O_12_	463.0875 (463.0871)	287.0537 [M+H-176]^+^	461.0723 (461.0725)	285.0405	+	+
21	9.75							ND	+
22	8.61	Apigenin-rutinoside isomer	C_27_H_30_O_14_	579.1706 (579.1708)	271.0591 [M+H-162-146]^+^	577.1561 (577.1563)	269.0455	+	ND
23	9.11	Apigenin-7-*O*-glucoside (Flavone Glycosides)	C_21_H_20_O_10_	433.1131 (433.1129)	271.0580 [M+H-162]^+^	431.0987 (431.0984)	269.0441	+	+
24	9.23	Apigenin 7-*O*-glucuronide	C_21_H_18_O_11_	447.0919 (447.0922)	271.0595 [M+H-176]^+^	445.0775 (445.0776)	269.0435	+	+
25	9.41	Salvianolic acid K	C_27_H_24_O_13_			555.1147 (555.1144)	359.0738, 161.0231, 135.0439	+	+
26	9.5	Chrysoeriol 7-glucuronide	C_22_H_20_O_12_	477.1031 (477.1028)	301.0711	475.0885 (487.0882)	299.0565	+	_+_
27	9.6	Rosmarinic acid	C_18_H_16_O_8_	361.0920 (361.0918)	163.0390 [M+H-C_9_H_10_O_5_]^+^ or [caffeic acid-H-H_2_O]^+^; 135.0441 [caffeic acid-H- H_2_O-CO]^+^	359.0774 (359.0772)	197.0456 [M-H-162]^−^; 179.0352 [M-H-180]^−^; 161.0247 [M-H- C_9_H_10_O_5_]^−^	+	+
28	10.3	Lithospermic acid B	C_27_H_22_O_12_	539.1190 (539.1188)	-	537.1041 (537.1038)	493.1137 [M-H-CO_2_]; 313.0718 [M-H-CO_2_-180]^−^ 295.0610 [M-H-CO_2_-198]^−^ 135.0455	+	+
29	11.7	Tetramethoxyflavone	C_19_H_18_O_8_	375.1071 (375.1074)	177.0550	373.0929 (373.0929)	197.0457, 175.0402, 135.0455	+	+
30	12.2	*Horminone*	C_20_H_28_O_4_	-	-	331.1916 (331.1915)	-	+	ND
31	12.3	Luteolin	C_15_H_10_O_6_	287.0555 (287.055)	153.0176 (C_7_H_5_O_4_) 135.0421 (RDA fragments)	285.0408 (285.0405)	-	+	+
32	13.3	Methoxy- coumarin	C_10_H_8_O_3_	177.0547 (177.0546)	163.0391, 145.0286, 117.0335, 89.0390	-	-	+	+
33	13.6	Salvianolic acid B	C_36_H_30_O_16_	719.1602 (719.1607)		717.1462 (717.1461)		+	ND
34	13.91	Apigenin (Flavone)	C_15_H_10_O_5_	271.0603 (271.0601)		269.0451 (269.0455)		+	+
35	14.3	Hispidulin (Flavone)	C_16_H_12_O_6_	301.0698 (301.0707)	286.0499	299.0565 (299.0561)	284.0311 [M-H-GluA-CH_3_]^−^	ND	+
36	14.41	3′,4′,5-Trihydroxy-7-methoxyflavone	C_16_H_12_O_6_	301.0710 (301.0707)	286.0471			ND	+
37	14.54	Jaceosidin (trihydroxyflavone)	C_17_H_14_O_7_	331.0815 (331.0812)	315.0517	329.0657 (329.0667)	314.0328	+	+
38	14.6	Viscosine	C_17_H_14_O_7_	331.0801 (331.0812)	315.0498	329.0617 (329.0667)	-	+	+
39	15.2	Rosmanol	C_20_H_26_O_5_	-	-	345.1708 (345.1707)	301.1808	+	ND
40	15.9	Dihydroxy-dimethoxyflavone	C_17_H_14_O_6_	315.0860 (315.0863)	254.0569	-	-	ND	+
41	16.54							ND	+
42	15.83	Hydroxycarnosic acid	C_20_H_28_O_5_	-	-	347.1859 (347.1864)	-	+	ND
43	16.07	Cirsimaritin	C_17_H_14_O_6_	315.0861 (315.0863)	-	313.0715 (313.0718)	298.0481 [M-H-CH_3_]; 283.0235 [M-H-CH_3_-CH_3_]^−^	+	+
44	16.3	Eupatorin (3′,5-Dihydroxy-4′,6,7-tri methoxyflavone)	C_18_H_16_O_7_	345.0971 (345.0969)	330.0655 [M+H-15]^+^, 312.0599 [M+H-15-18]^+^, 269.0411 [M+H-15-18-28-15]^+^	-	-	+	+
45	17.36	Genkwanin (Flavone)	C_16_H_12_O_5_	285.0760 (285.0757)	242.0569	283.0613 (283.0612)	268.0384	+	+
46	17.7	Pectolinarigenin (Dihydroxy-dimethoxyflavone)	C_17_H_14_O_6_	315.0869 (315.0863)	300.0624 [M+H-15]^+^; 282.0522 [M+H-15-18]^+^	313.0718 (313.0718)	298.0470 283.0233 255.0300	t	+
47	18.3	Carnosic acid	C_20_H_28_O_4_	333.2061 (333.2060)	315.1958 [M+H-H_2_O]^+^ 287.2008	331.1917 (331.1915)	-	+	ND
48	18.5	labdadien-olides	C_25_H_38_O_5_	441.2613 (441.2611) [M+Na]^+^	401.2689 [M+H-H_2_O]^+^	-	-	+	ND
49	18.9	Tormentic acid	C_30_H_48_O_5_			487.3435 (487.3429)	469.3301 [M-H-18]^−^	+	ND
50	19.2	Salvigenin (5-hydroxy-4′, 6, 7-trimethoxy flavone)	C_18_H_16_O_6_	329.1021 (329.1020)	296.0671 [M+H-15-18]^+^; 268.0735 [M+H-15-18-28]^+^	327.0875 (327.0874)	-	+	+
51	20.43	1,11,20-Trihydroxy-3-lupanone (1β,11α)-form	C_30_H_50_O_4_	-	-	475.3789 (475.3782)	-	+	ND
52	21.5	Maslinic acid	C_30_H_48_O_4_	-	-	471.3477 (471.3480)	417.3000	+	_+_
53	21.73	Hydroxyursolic acid	C_30_H_48_O_4_	473.3625 (473.3625)	455.3516 [M+H-H_2_O]^+^ 437.3402; 409.3484	471.3477 (471.348)	-	+	+
54	21.81	2α-Hydroxyursolic acid	C_30_H_48_O_4_	473.3625 (473.3625)	455.3516 [M+H-H_2_O]^+^	471.3481 (471.3480)	-	+	+
55	24.1	Ursolic acid/Oleanolic acid/3-Epiursolic acid	C_30_H_48_O_3_	457.3680 (457.3676)	439.3573 [M+H-H_2_O]^+^	455.3530 (455.3531)	-	+	ND
56	24.6							+	+
57	24.7							+	+

ND = Not detected; + detected.

## References

[1] Muyumba N.W., Mutombo S.C., Sheridan H., Nachtergael A., Duez P. (2021). Quality control of herbal drugs and preparations: The methods of analysis, their relevance and applications. Talanta Open.

[2] Sarsenbayev K.N., Egamberdieva D., Öztürk M. (2018). Chapter 10. Medicinally important plants of Kazakhstan. Vegetation of Central Asia and Environs.

[3] Zhusupova G.E., Litvinenko Y.A., Zhussupova A.I. (2018). Methodology for Processing Medicinal Plant Materials.

[4] Ghorbani A., Esmaeilizadeh M. (2017). Pharmacological properties of *Salvia officinalis* and its components. J. Tradit. Complement. Med..

[5] Aleksovski A., Sovova H. (2007). Supercritical CO_2_ extraction of *Salvia officinalis* L.. J. Supercrit. Fluids.

[6] Sa C., Ramos A., Azevedo M., Lima C., Fernandes-Ferreira M., Pereira-Wilson C. (2009). Sage tea drinking improves lipid profile and antioxidant defences in humans. Int. J. Mol. Sci..

[7] Lu Y., Yeap Foo L. (2001). Salvianolic acid L, a potent phenolic antioxidant from *Salvia officinalis*. Tetrahedron Lett..

[8] Hamidpour M., Hamidpour R., Hamidpour S., Shahlari M. (2014). Chemistry, pharmacology, and medicinal property of sage (*Salvia*) to prevent and cure illnesses such as obesity, diabetes, depression, dementia, lupus, autism, heart disease, and cancer. J. Tradit. Complement. Med..

[9] Ayoub I.M., George M.Y., Menze E.T., Mahmoud M., Botros M., Essam M., Ashmawy I., Shendi P., Hany A., Galal M. (2022). Insights into the neuroprotective effects of *Salvia officinalis* L. and *Salvia microphylla* Kunth in the memory impairment rat model. Food Funct..

[10] Christensen K.B., Jorgenson M., Kotowska D., Peterson R.K., Kristiansen K., Christensen L.P. (2010). Activation of the nuclear receptor PPARγ by metabolites isolated from sage (*Salvia officinalis* L.). J. Ethnopharmacol..

[11] Javid H., Moein S., Moein M. (2022). An investigation of the inhibitory effects of dichloromethane and methanol extracts of *Salvia macilenta, Salvia officinalis*, *Salvia santolinifola* and *Salvia mirzayanii* on diabetes marker enzymes, an approach for the treatment diabetes. Clin. Phytosci..

[12] el Hadri A., Gomez del Rio M.A., Sanz J., Coloma A.G., Idaomar M., Ribas Ozanas B., Benedí González J., Sánchez Reus M.I. (2010). Cytotoxic activity of α-humulene and transcaryo-phyllene from *Salvia officinalis* in animal and human tumor cells. An. R. Acad. Nac. Farm..

[13] Privitera G., Luca T., Castorina S., Passanisi R., Ruberto G., Napoli E. (2019). Anticancer activity of *Salvia officinalis* essential oil and its principal constituents against hormone-dependent tumour cells. Asian Pac. J. Trop. Biomed..

[14] Loizzo M.R., Tundis R., Menichini F., Saab A.M., Statti G.A., Menichini F. (2007). Cytotoxic activity of essential oils from Labiatae and Lauraceae families against in vitro human tumor models. Anticancer Res..

[15] Capek P., Hríbalová V. (2004). Water-soluble polysaccharides from *Salvia officinalis* L. possessing immunomodulatory activity. Phytochemistry.

[16] Bonesi M., Loizzo M.R., Acquaviva R., Malfa G.A., Aiello F., Tundis R. (2017). Anti-inflammatory and antioxidant agents from *Salvia* genus (Lamiaceae): An assessment of the current state of knowledge. Med. Chem..

[17] Bekut M., Brkic S., Kladar N., Dragovic G., Gavaric N., Bozin B. (2018). Potential of selected Lamiaceae plants in anti (retro) viral therapy. Pharmacol. Res..

[18] Kylyshbaeva G.B., Bozshataeva G.T., Ospanova G.S. (2013). Study of biologically active compounds in species of the genus *Salvia* (*Salvia* L., Lamiaceae) in the conditions of the South Kazakhstan region. Int. J. Appl. Fund. Res..

[19] Cui N., Chen T., Liao B., Xu J., Li X. (2021). The biology of medicinal resource substitution in *Salvia*. Chin. Med..

[20] Wu Y.-B., Ni Z.-Y., Shi Q.-W., Dong M., Kiyota H., Gu Y.-C., Cong B. (2012). Constituents from *Salvia* species and their biological activities. Chem. Rev..

[21] Vosoughi N., Gomarian M., Ghasemi Pirbalouti A., Khaghani S., Malekpoor F. (2018). Essential oil composition and total phenolic, flavonoid contents, and antioxidant activity of sage (*Salvia officinalis* L.) extract under chitosan application and irrigation frequencies. Ind. Crops Prod..

[22] Pourhosseini M., Asgarpanah J. (2015). Essential and fixed oil chemical profiles of *Salvia aegyptiaca* L. flowers and seeds. J. Chil. Chem. Soc..

[23] Kasimu R., Wang X., Wang X., Hu J., Wang X., Mu Y. (2018). Antithrombotic effects and related mechanisms of *Salvia deserta* Schang root EtOAc extracts. Sci. Rep..

[24] Li M., Li Q., Zhang C. (2013). An ethnopharmacological investigation of medicinal *Salvia* plants (Lamiaceae) in China. Acta Pharm. Sin. B.

[25] Zheng X., Kadir A., Zheng G., Jin P., Qin D., Maiwulanjiang M., Aisa H.A., Yao G. (2020). Antiproliferative abietane quinone diterpenoids from the roots of *Salvia deserta*. Bioorg. Chem..

[26] Llurba-Montesino N., Schmidt T.J. (2018). *Salvia* species as sources of natural products with antiprotozoal activity. Int. J. Mol. Sci..

[27] Aćimović M., Kiprovski B., Rat M., Sikora V., Popović V., Koren A., Brdar-Jokanović M. (2018). *Salvia sclarea*: Chemical composition and biological activity. J. Agron. Technol. Eng. Manag..

[28] Yalcin H., Ozturk I., Tulukcu E., Sagdic O. (2011). Effect of γ-irradiation on bioactivity, fatty acid compositions and volatile compounds of clary sage seed (*Salvia sclarea* L.). J. Food Sci..

[29] Wong J., Chiang Y.F., Shih Y.H., Chiu C.-H., Chen H.-Y., Shieh T.-M., Wang K.-L., Huang T.-C., Hong Y.-H., Hsia S.-M. (2020). *Salvia sclarea* L. essential oil extract and its antioxidative phytochemical sclareol inhibit oxytocin-induced uterine hypercontraction dysmenorrhea model by inhibiting the Ca^2+^-MLCK-MLC20 signaling cascade: An ex vivo and in vivo study. Antioxidants.

[30] Mahboubi M. (2020). Clary sage essential oil and its biological activities. Adv. Tradit. Med..

[31] Cui H., Zhang X., Zhou H., Zhao C., Lin L. (2015). Antimicrobial activity and mechanisms of *Salvia sclarea* essential oil. Bot. Stud..

[32] Jasicka-Misiak I., Poliwoda A., Petecka M., Buslovych O., Shlyapnikov V., Wieczorek P.P. (2018). Antioxidant phenolic compounds in *Salvia officinalis* L. and *Salvia sclarea* L.. Ecol. Chem. Eng. S.

[33] (2008). State Pharmacopeia of the Republic of Kazakhstan.

[34] (2009). State Pharmacopeia of the Republic of Kazakhstan.

[35] (2014). State Pharmacopeia of the Republic of Kazakhstan.

[36] Pagare S., Bhatia M., Tripathi N., Pagare S., Bansal Y.K. (2015). Secondary metabolites of plants and their role: Overview. Curr. Trends Biotechnol. Pharm..

[37] Yahfoufi N., Alsadi N., Jambi M., Matar C. (2018). The immunomodulatory and anti-inflammatory role of polyphenols. Nutrients.

[38] Jantan I., Ahmad W., Bukhari S.N.A. (2015). Plant-derived immunomodulators: An insight on their preclinical evaluation and clinical trials. Front. Plant Sci..

[39] Samec M., Liskova A., Koklesova L., Mathews S., Radovan S., Pavol M. (2020). The role of plant—Derived natural substances as immunomodulatory agents in carcinogenesis. J. Cancer Res. Clin. Oncol..

[40] Wong C.K., Bao Y.X., Wong E.L., Leung P.C., Fung K.P., Lam C.W. (2005). Immunomodulatory activities of Yunzhi and Danshen in post-treatment breast cancer patients. Am. J. Chin. Med..

[41] Wong C.K., Tse P.S., Wong E.L., Leung P.C., Fung K.P., Lam C.W. (2004). Immunomodulatory effects of yun zhi and danshen capsules in health subjects—A randomized, double-blind, placebo-controlled, crossover study. Int. Immunopharmacol..

[42] Moon S., Shin S., Kim S., Oh H.E., Han S., Lee S., Kim K. (2011). Role of *Salvia miltiorrhiza* for modulation of Th2-derived cytokines in the resolution of inflammation. Immune Netw..

[43] Kang B.Y., Chung S.W., Kim S.H., Ryu S.Y., Kim T.S. (2000). Inhibition of interleukin-12 and interferon-gamma production in immune cells by tanshinones from *Salvia miltiorrhiza*. Immunopharmacology.

[44] Shin J., Kim O.K., Kim S., Bae D., Lee J., Park J., Jun W. (2020). Immunomodulatory effect of a *Salvia plebeian* R. aqueous extract in forced swimming exercise-induced mice. Nutrients.

[45] Zhang J.M., An J. (2007). Cytokines, inflammation, and pain. Int. Anesthesiol. Clin..

[46] Yin M., Zhang Y., Li H. (2019). Advances in research on immunoregulation of macrophages by plant polysaccharides. Front. Immunol..

[47] Im S.A., Lee Y.R., Lee Y.H., Oh S.T., Gerelchuluun T., Kim B.H., Kim Y., Yun Y.P., Song S., Lee C.K. (2007). Synergistic activation of monocytes by polysaccharides isolated from *Salicornia herbacea* and interferon-gamma. J. Ethnopharmacol..

[48] Han C., Yang J., Song P., Wang X., Shi W. (2018). Effects of *Salvia miltiorrhiza* polysaccharides on lipopolysaccharide-induced inflammatory factor release in RAW264.7 Cells. J. Interferon Cytokine Res..

[49] Wang H., Ma C., Sun-Waterhouse D., Geoffrey W., Waterhouse J.I.N., Kang W. (2022). Imunoregulatory polysaccharides from *Apocynum venetum* L. flowers stimulate phagocytosis and cytokine expression via activating the NF-κB/MAPK signaling pathways in RAW264.7 cells. Food Sci. Hum. Wellness.

[50] Cui L., Chen L., Yang G., Li Y., Qiao Z., Liu Y., Meng Y., Zhou Y., Sun L. (2021). Structural characterization and immunomodulatory activity of a heterogalactan from Panax ginseng flowers. Food Res. Int..

[51] Cheng X.Q., Li H., Yue X.L., Xie J.Y., Zhang Y.Y., Di H.Y., Chen D.F. (2010). Macrophage immunomodulatory activity of the polysaccharides from the roots of Bupleurum smithii var. parvifolium. J. Ethnopharmacol..

[52] Huston D.P. (1997). The biology of the immune system. JAMA.

[53] Chen Y., Li H., Li M., Niu S., Wang J., Shao H., Li T., Wang H. (2017). *Salvia miltiorrhiza* polysaccharide activates T Lymphocytes of cancer patients through activation of TLRs mediated-MAPK and -NF-κB signaling pathways. J. Ethnopharmacol..

[54] Medina-Torres N., Ayora-Talavera T., Espinosa-Andrews H., Sánchez-Contreras A., Pacheco N. (2017). Ultrasound assisted extraction for the recovery of phenolic compounds from vegetable sources. Agronomy.

[55] Shekar H.S., Rajamma A.J., Sateesha S.B. (2017). Application of ultrasound to pharmaceutical industry: An overview. J. Pharm. Drug Deliv. Res..

[56] Hemingway R.W., Karchesy J.J. (2012). Chemistry and Significance of Condensed Tannins.

[57] Avula B., Bae J.Y., Chittiboyina A.G., Wang Y.H., Wang M., Srivedavyasasri R., Ali Z., Li J., Wu C., Khan I.A. (2022). Comparative analysis of five *Salvia species* using LC-DAD-QToF. J. Pharm. Biomed. Anal..

[58] National Research Council (1996). Guide for the Care and Use of Laboratory Animals.

[59] Zhang X., Goncalves R., Mosser D.M. (2008). The isolation and characterization of murine macrophages. Curr. Protoc. Immunol..

[60] Lim J.F., Berger H., Su I.H. (2016). Isolation and activation of murine lymphocytes. J. Vis. Exp..

[61] Wysocki L.J., Sato V.L. (1978). “Panning” for lymphocytes: A method for cell selection. Proc. Natl. Acad. Sci. USA.

